# Study protocol: pragmatic randomized control trial of my tools 4 care- in care (MT4C-in care) a web-based tool for family Carers of persons with dementia residing in long term care

**DOI:** 10.1186/s12877-020-01690-w

**Published:** 2020-08-10

**Authors:** Wendy Duggleby, Hannah O’Rourke, Jennifer Swindle, Shelley Peacock, Carrie McAiney, Pamela Baxter, Genevieve Thompson, Véronique Dubé, Cheryl Nekolaichuk, Sunita Ghosh, Jayna Holroyd-Leduc

**Affiliations:** 1 Faculty of Nursing, University of Alberta, Level 3 Edmonton Clinic Health Academy, 11405 87 Avenue, Edmonton, Alberta T6G 1C9 Canada; 2 College of Nursing, University of Saskatchewan, 104 Clinic Place, Saskatoon, SK S7N 2Z4 Canada; 3 Schlegel-UW Research Institute for Aging, School of Public Health and Health Systems, University of Waterloo, 200 University Avenue West, Waterloo, ON N2L 3G1 Canada; 4 School of Nursing, McMaster University, 1280 Main St. W, Hamilton, ON L8S 4K1 Canada; 5 College of Nursing, Faculty of Health Sciences, University of Manitoba, 89 Curry Place, Winnipeg, MB R3T 2N2 Canada; 6 Faculty of Nursing, Université de Montréal Marguerite d’Youville Research, Université de Montréal, PO Box 6128, Centre-ville Station, Montréal, QC H3C 3J7 Canada; 7 Department of Oncology, University of Alberta, c/o Palliative Institute, DC 404, 1090 Youville Drive, Edmonton, AB T6L 0A3 Canada; 8 Departments of Medical Oncology and Mathematical and Statistical Sciences, University of Alberta, Edmonton, Canada; 9 Alberta Health Services-Cancer Control, 0058 Cross Cancer Institute, 11560 University Ave, Edmonton, AB T6G 1Z2 Canada; 10 Division of Geriatric Medicine, Departments of Medicine and Community Health Sciences, University of Calgary, 1403 – 29th Street NW, Calgary, AB T2N2T9 Canada

**Keywords:** Carers, Dementia, Pragmatic trial, Online intervention, Long term care

## Abstract

**Background:**

When a family member resides in long term care facility (LTC), family carers continue caregiving and have been found to have decreases in mental health. The aim of My Tools 4 Care – In Care (an online intervention) is to support carers of persons living with dementia residing in LTC through transitions and increase their self-efficacy, hope, social support and mental health. This article comprises the protocol for a study to evaluate My Tools 4 Care-In Care (MT4C-In Care) by asking the following research questions:

1. Is there a 2 month (immediately post-intervention) and 4 month (2 months post-intervention) increase in mental health, general self-efficacy, social support and hope, and decrease in grief and loneliness, in carers of a person living with dementia residing in LTC using MT4C-In CARE compared to an educational control group?
2. Do carers of persons living with dementia residing in LTC perceive My Tools 4 Care- In Care helps them with the transitions they experience?

**Methods:**

This study is a single blinded pragmatic mixed methods randomized controlled trial. Approximately 280 family carers of older persons (65 years of age and older) with dementia residing in LTC will be recruited for this study. Data will be collected at three time points: baseline, 2 month, and 4 months. Based on the feasibility study, *we hypothesize that participants using MT4C-In Care will report significant increases in hope, general self-efficacy, social support and mental health quality of life, and significant decreases in grief and loneliness from baseline, as compared to an educational control group*. To determine differences between groups and over time, generalized estimating equations analysis will be used. Qualitative descriptive analysis will be used to further evaluate MT4C-In Care and if it supports carers through transitions.

**Discussion:**

Data collection will begin in four Canadian provinces (Alberta, Manitoba, Ontario and Saskatchewan) in February 2020 and is expected to be completed in June 2021. The results will inform policy and practice as MT4C-In Care can be revised for local contexts and posted on websites such as those hosted by the Alzheimer Society of Canada.

**Trial registration:**

NCT04226872 ClinicalTrials.gov Registered 09 January 2020

Protocol Version #2 Feb 19, 2020.

## Background

In 2018 in Canada, 564,000 people were living with dementia, and this number is expected to almost double by 2031 [1]. Dementia affects not only the person living with the disease, but their families, friends, and communities. In particular, family and friend carers (hereafter referred to as ‘carers’) are challenged to cope with the many transitions that occur across all stages of dementia. For these carers, transitions involve learning new tasks, making significant adaptations to the physical and/or social environment, and changing their roles and relationships [2]. Although carers have positive experiences as a result of these significant changes, many carers experience significant physical and mental health challenges, including distress and burnout [3].

Little is known about the transition experience, support needs, and effective interventions for carers after their family member with dementia moves to a 24-h long-term care (LTC) community [4, 5]. Caregiving continues when family members of persons living with dementia reside in LTC; in fact, this time period is often characterised by continued involvement, during which carers must learn multiple new roles and make significant and often stressful adjustments [4–6]. Carers of a family member living with dementia in a LTC community are an at-risk group**,** and evidence suggests that carers’ mental health may actually worsen after their relative with dementia moves to LTC [7]. Carers may report feelings of blame, self-doubt, loneliness, isolation, and powerlessness [4, 7] which negatively affect their mental health [8]. In contrast, hope, confidence in the ability to deal with difficult situations or self-efficacy, and social support can improve their mental health.

Transitions are significant changes experienced by an individual that are incorporated into their lives [9]. Carers of older persons living with dementia residing in LTC experience multiple complex transitions, such as changes in roles and relationships, physical and mental health, and hope [2]. Social support influences feelings of loneliness and mental health [10], and may improve one’s ability to cope with stress during times of transition [11]. Social support is a function of a person’s social network, and may be instrumental (e.g., tangible services), emotional (e.g., expressions of empathy), informational (e.g., advice) or appraisal support (e.g., information useful for self-evaluation) [12]. Interventions that support carers in dealing with transitions are essential for their quality of life.

Due to the costs, and sometimes limited accessibility associated with face-to-face interventions, there is a shift towards technology-driven interventions to support carers [13–15]. Three reviews have been published on web-based interventions for carers of persons living with dementia [13–15]. Boots et al. [14] in their review of 12 studies concluded that multi-component online interventions that were flexible and tailored to the individual resulted in increased carer well-being. These conclusions are similar to those of reviews of online interventions for carers of people living with a variety of diagnoses, including dementia [16–18]. The reviewed studies suggest that web-based interventions show promise in increasing the mental health of carers. However, none of the reviewed interventions were specifically tailored for carers of persons living with dementia in the context of LTC.

Two multi-component web-based interventions to support carers of persons living with dementia have been developed by our research team. They are based on Meleis’ transition theory [19] and combine information and interactive activities tailored to carers of persons living with dementia. The first intervention (My Tools 4 Care; https://www.mytools4care.ca) was developed for carers of persons living with dementia in the community [14]. My Tools 4 Care was recently evaluated using a pragmatic randomized control trial [20] and had a significant positive impact on participants’ hope, as compared to an educational control group. Hope has also been found to have a significant positive relationship with mental health in this population [2].

Carers of older persons living with dementia residing in LTC approached us to adapt My Tools 4 Care for their use. The experiences of carers of persons living with dementia in the community and those in LTC appear to share some similarities. For example, carers of persons living with dementia residing in LTC continue to experience loss [21] and significant changes in hope [22]. However, many differences are also apparent. For example, negative interactions between carers and LTC staff and poor perceptions of care have a negative impact on carers [7, 23], potentially resulting in an increased need for healthcare services. Moreover, carers in LTC are concerned about end-of-life decision-making [16].

Working with carers, the research team developed My Tools 4 Care-In Care (MT4C-In Care; https://www.mytools4careincare.ca) for use with carers of a person with dementia residing in LTC. MT4C-In Care is a self-administered, multicomponent, flexible, and interactive website. MT4C-In Care aims to support carers of persons living with dementia to adapt to the significant transitions they face after their relative has moved to a LTC setting by increasing hope, self-efficacy, and social support, and decreasing grief and loneliness. Throughout the website, the user is prompted to mobilize their existing support networks to obtain social support (e.g., identifying people who give one strength or discussing carer goals with facility staff). As a resource provided by the carer’s social network (e.g., the local Alzheimer Societies), MT4C-In Care also delivers support directly. For example, MT4C-In Care offers emotional support (e.g., statements aimed to validate feelings of guilt), information support (e.g., fact sheet on ways to address loneliness), and appraisal support (e.g., prompts to reflect on experiences as a carer).

**Fig. 1:**
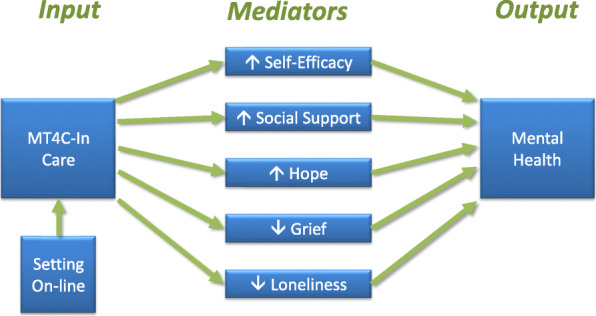
Conceptual Framework for MT4C-In Care. Conceptual framework of the proposed mediators (proximal outcomes) and primary (distal) outcome of MT4C-In Care

A feasibility study of MT4C-In Care with 37 participants demonstrated an increase in hope (*p* = 0.006) and decrease in grief (*p* = 0.005) from baseline to a time point 2 months later [24]. Although statistically significant differences in mental health were not detected, hope (r = 0.43, *p* = 0.027) and grief (r = − 0.66, *p* = 0.00) were significantly related to mental health. Whether social support and loneliness mediate the effects of MT4C-In Care upon mental health has not been evaluated to date. Prior to this pragmatic trial, we conducted focus groups with carers in four Canadian provinces to optimize the tool’s ability to improve carers’ perceptions of social support. Based on the feasibility study and focus groups, below is a diagram of the MT4C-In Care framework (Fig. 1) in which we hypothesize that MT4C-In Care increases hope, general self-efficacy and social support, and decreases grief and loneliness. Through these mechanisms, the tool increases mental health of carers of older persons living with dementia residing in LTC.

### Purpose

The purpose of this study is to evaluate whether use of MT4C-In Care over 2 months is effective to decrease loneliness and grief, and increase hope, general self-efficacy, social support and mental health as compared to an educational control group. It will address the following research questions:

1. Is there a 2 month (immediately post-intervention) and 4 month (2 months post-intervention) increase in mental health, general self-efficacy, social support and hope, and decrease in grief and loneliness, in carers of a person living with dementia residing in LTC using MT4C-In CARE compared to an educational control group?
2. Do carers of persons living with dementia residing in LTC perceive My Tools 4 Care- In Care helps them with the transitions they experience?

## Methods/design

### Study design

This pragmatic mixed methods randomized controlled trial is longitudinal, multi-site, and involves repeated measures. Participants are randomly assigned to an intervention or control group using REDCap, a secure, password-protected web application housed by the University of Alberta. The intervention group receives an educational resource and access to MT4C-In Care over a two-month period. The educational control group also receives the educational resource but will not be granted access to MT4C-In Care until the study period is over. Recruitment and data collection are planned between February 2020 and June 2021 in four provinces (Alberta, Saskatchewan, Manitoba and Ontario). Outcome data collection will occur at baseline, and 2 and 4 months from the start of the intervention. Process data collected at 2 and 4 months include qualitative data related to participant perceptions of MT4C-In Care and quantitative data related to frequency of use. Quantitative and qualitative data related to study processes (e.g., frequency and reasons for missing data) will be collected concurrently throughout the study. The qualitative data will help to explain the quantitative findings.

Reporting of this protocol followed the SPIRIT (Standard protocol items: recommendations for interventional trials) [25] guidelines for reporting protocols (SPIRIT checklist in supplementary files). This study received ethical approval from the University of Alberta Health Research Ethics Board (#Pro00090771), University of Saskatchewan Research Ethics Board (#1385), Education/Nursing Research Ethics Board at the University of Manitoba (E2019:127), and the Hamilton Integrated Research Ethics Board (#7659). If there are any changes in the protocol an amendment will be sent to the respective ethics boards for approval.

### Participants/settings

Carer is defined broadly as unpaid family or friends who provide any form of care to someone living with dementia. This care can include, but is not limited to, assisting with personal care, taking food, arranging appointments, and advocating for their relative. Carers will meet the following inclusion criteria: 1) English-speaking; 2) ≥ 18 years of age; 3) provides physical, emotional, or financial care to persons living with dementia who is ≥65 years of age and lives in a LTC home; and 4) has an email address and access to a computer with internet. Exclusion criteria are: 1) care partner is no longer living; 2) care partner resides in the community; and 3) care partner is currently hospitalized or in an assisted living facility.

Participants in four provinces in Canada (Alberta, Saskatchewan, Manitoba and Ontario) will be asked to participate in the study via telephone. The setting will be of their choosing, so that they can participate from their homes, or wherever they feel most comfortable.

### Recruitment and randomization

Advertisements in local community newspapers which direct potential participants to contact the research coordinators (one in each province) via email or a toll-free number will be used to recruit participants. As well, LTC facilities and the Alzheimer Societies of Brant, Haldimand Norfolk, Hamilton Halton, Manitoba, Saskatchewan, Alberta/North West Territories, and Calgary, will assist with recruiting potential participants.

Research assistants will contact potential participants in each province via telephone to explain the study, answer any questions, and obtain informed verbal consent to participate. A tracking sheet of the numbers of potential participants who are approached to participate in the study, as well as the number of eligible consenters and non-consenters will be kept in a secure shared drive separate from the data. Reasons for non-consent will be documented.

A unique study identifier will be assigned to each participant to maintain confidentiality. REDCap will be used to randomly assign (1:1 ratio) participants to either the intervention or control group. Participants will be blinded to assignment status, and will be told that they will be offered one of two ways of providing support. In order for blinding to occur, the control group will be an educational control group receiving educational information. Two versions of the consent forms will be used one for the intervention and another for the control group (both versions of the consent forms are in the supplementary file). The RAs will read to the participants the version of the informed consent that will describe the study and group allocation procedures specific to their assigned group. Following the first interview, the informed consent will be emailed to participants. Data collection will then proceed with gathering demographic data and the baseline survey measures. Immediately following the baseline interview, all participants will be sent a copy of the appropriate consent form by email. Un-blinding will occur upon the request of the participant.

### Educational control group

The educational resource that will be emailed to the control group participants is an Alzheimer Society document called *Progression* [26] immediately following baseline interviews*.* This document was developed for use by people with dementia and their family and friend carers. It provides a summary overview of the stages of the disease, importance of person-centred care, prompts to access support, and a list of additional resources. It is available for free on the Alzheimer Society of Canada website. Following data collection, control group participants will also be granted access to MT4C-In Care.

### Intervention: my tools 4 care-in care

The intervention group will receive via email the educational resource provided to the control group and instructions on how to access to the MT4C-In Care site immediately following baseline interviews. MT4C-In Care access will occur for 2 months via an assigned web link and unique username and password. Participants will access the MT4C-In Care tool as frequently as desired, and will choose which content to engage with. They may choose not to access MT4C-In Care at any time. Some activities prompt the participants to enter information into the site, and this information will not be accessed by anyone, including the research team. The components of MT4C-In Care include a home page which introduces the toolkit and sections: a) “About Me” (contains guided evidence-based activities), b) “Common Changes to Expect”, c) “Frequently Asked Questions”, and d) Resources. More detailed information about My Tools 4 Care-In Care is published elsewhere [24].

### Measures

#### Carer demographic form

A demographic form will be used to collect data from carers such as age, gender, language, relationship to the person living with dementia. Carers will also provide information about the person living with dementia including their stage of disease, age, gender, and length of time in LTC. A similar demographic form was used in the pragmatic trial of MT4C [20].

#### Process measures

Several process measures will be used throughout the study. Research assistants will track all participants’ participation in the study, including recruitment rates, and reasons for study withdrawal and missing data. The other measures include the MT4C-In Care checklist and qualitative interviews.

#### MT4C-in care checklist

MT4C-In Care checklist will be used to collect data on access and use of MT4C-In Care. Data will be collected at 2 months following the start of the intervention. Participants in the intervention group will be asked to complete the MT4C-In Care self-report checklist during the interview. The questions include: 1) how often they use MT4C-In Care, b) the total time spent on MT4C-In Care and c) content accessed over the past 2 months. They will also be asked three dichotomous questions about whether MT4C-In Care improved their knowledge and skills, mental health, and well-being, from their perspective. As well data will be collected on the utilization of other caregiver support websites and support groups. There will be no prohibited interventions during this study. A copy of the checklist can be found in the supplementary files.

#### Qualitative interviews

At 2 months, a sub-sample of 40 participants who received the intervention will be interviewed over the phone using a semi-structured interview guide. The nature of the questions will focus on participants’ experiences when working on MT4C-In Care, if it helped them deal with their significant changes, and what did they liked best and least. The qualitative interview guide was developed for this study and is found in the article’s supplementary files. The purpose of the interview will be to understand their perceptions of MT4C-In Care in more depth.

Based on our previous study [27], the sample should contain approximately 15 participants from each of Alberta and Ontario and 5 participants from each of Saskatchewan and Manitoba, or enough to obtain rich findings. Interviews will last between 15 and 30 min and will be audiotaped. The research assistant will record field notes that reflect the tone of the interview, any difficulties faced (e.g., multiple interruptions or feeling rushed), and initial thoughts related to salient points raised.

#### Outcome measures

The **Mental Component Summary (MCS) score**, short form Health Survey (SF-12v2) [28], is the primary outcome because MT4C-In Care aims, ultimately, to support carers’ psychological well-being [29]. The higher the score on the MCS, the higher the mental health. The **SF-12v2** measures perceived function and well-being with 12 items using a variety of Likert scales. A majority of carers are older adults, and the SF-12v2 has been used with community-dwelling older adults. The tool’s eight domains (physical functioning, role-physical, bodily pain, general health, vitality, social functioning, roles, and emotional and mental health) can be combined to derive scores (maximum 100) for a physical component summary (PCS) and mental component summary (MCS). The SF-12v2 is used widely and in a variety of populations, and evidence supports its internal consistency, reliability, and construct validity [30, 31].

We selected as secondary outcomes those variables which are the hypothesized mechanisms by which MT4C-In Care affects mental health including hope, general self-efficacy, social support, grief and loneliness. Therefore, these are the proposed proximal outcomes targeted by MT4C-In Care. Measures of hope, self-efficacy, grief, loneliness, and social support are:

1. The **Herth Hope Index (HHI)**: 12 items measure three dimensions of hope (temporality and future, positive readiness and expectancy, and interconnectedness [32]). Each item is rated with a 4-point Likert scale. Scores range from 12 to 48 (higher scores indicate more hope). The measure has been found to be a reliable and valid measure with carers [33, 34].
2. The **General Self-Efficacy Scale (GSES):** 10 items measure ability to handle adversity, each rated with a 4-point Likert scale [35]. Scores range from 10 to 40 (higher scores indicate more self-efficacy). Evidence supports the measure’s reliability and validity across populations [36].
3. The **Non-Death Version Revised Grief Experience Inventory (NDRGEI):** 22 items measure grief of people who are not bereaved, each rated with a 6-point Likert scale [37]. The scale has just two items that are different from the version of the scale used with people that are bereaved. Four domains are measured, including existential concerns, depression, guilt and distress. Scores range from 22 to 132 (higher scores indicate more grief and loss). Evidence supports the scale’s reliability and validity [36].
4. The **Three-Item Loneliness Scale:** 3 items measure perceptions of companionship, each rated with a 3-point Likert scale [38]. Scores range from 3 to 9 (higher scores indicate more loneliness). The measure is based on items from the revised University of California Los Angeles loneliness scale, but contains fewer items, takes less than 2 min to complete, and was selected for the current study to prevent respondent burden. It is validated for use with older adults. Evidence supports its reliability, and convergent and discriminant validity [39].
5. The **Multidimensional Scale of Perceived Social Support (MSPSS):** 12 items measure social support, each rated with a 7-point Likert scale [39]. Three subscales can be calculated, for significant others, family, and friends. The total score is an average, calculated by summing all items (which could range from 12 to 84) and dividing by 12 (higher scores indicate more social support). The measure is used widely and across populations. Evidence supports its internal and test-retest reliability, validity, and three-factor structure [40, 41].

### Power and sample size

We aim to recruit a convenience sample of 280 participants, 140 in each of the intervention and control groups. Our design for the outcome analysis includes three repeated measurements to observe how the change in outcomes over time differs between the intervention and control group. This sample size achieves 89% power to detect a 15% difference in the mental health summary score between the two groups over time, when alpha level is 5%. This calculation assumed a Compound Symmetry covariance structure, a proportion of 30% in group 2, and correlation between observations on the same subject of 0.50.

### Data collection

Data will be collected during audiotaped telephone interviews by trained, site-specific research assistants (data collectors) at three time periods: baseline, 2 months and 4 months. The qualitative interviews will be conducted at 2 months. Data collectors will obtain verbal consent to participate in the study prior to each telephone interview. Table 1 outlines the data collection for this study using the SPIRIT table format.

**Table 1:**
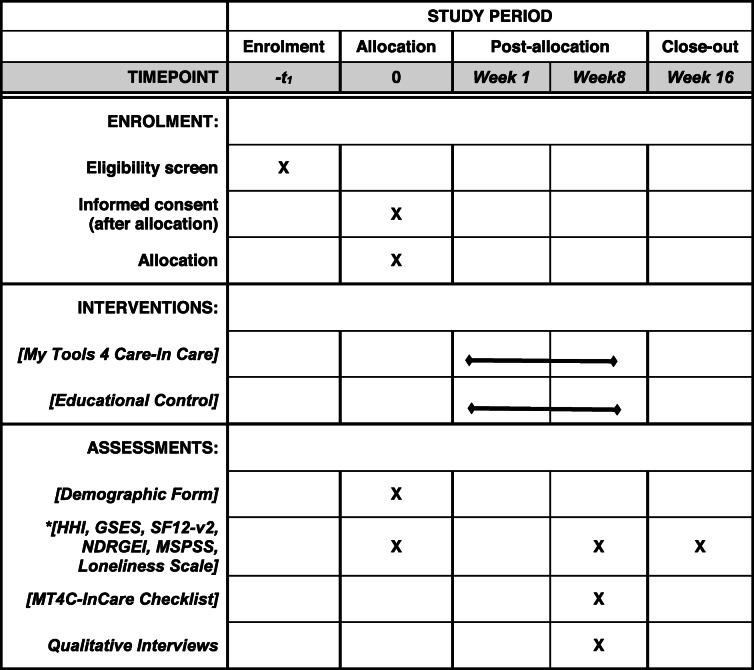
Schedule of Enrolment, Intervention (MT4C-In Care), and Assessments

*HHI (Herth Hope Index), GSES (General Self Efficacy Scale), SF12-v2 (Short Form 12-version 2), NDRGEI (Non-Death Revised Grief Experience Inventory), MSPSS (Multi-dimensional Scale of Perceived Social Support)

Using participants’ email addresses, a study brochure with the research coordinator’s contact information, and a copy of all study questionnaires (watermarked “for reference only”), will be sent to all participants after initial contact. Participants may use the “for reference only” questionnaires as a resource when data collectors are asking them for a verbal response to the questions during the telephone interviews. All contact with participants, including email communication, will be tracked by the data collectors on a data collection tracking sheet. Two weeks prior to each scheduled interview, a reminder will be sent to the participants to promote study retention. If the participant requests a change in the date and time of an interview, or misses a scheduled interview, the interview will be re-scheduled within approximately 1 week before or after the interview due date.

Data collectors will be trained before the study begins and will meet every 2 weeks during the study to discuss issues and challenges with data collection in order to maintain reliability and validity of data. As well data collectors will be reminded at each meeting to report any adverse events from the trial as soon as it occurs. A resource list has been developed to provide to distressed caregivers during the study by data collectors and they will encourage caregivers to contact their family physicians if needed.

Data will also be reviewed monthly by a working group comprised of the provincial PIs, data collectors and a statistician to identify any concerns and make any necessary changes. Interim analysis will occur every 6 months and presented at research team meetings for discussion. At that time discussion will occur if there is any reason to terminate the trial based on the analysis. Because the intervention is minimal risk to participants the research team will function as a Data Monitoring Committee.

### Data management plan

All data collectors (i.e., research assistants) will login to REDCap (a secure web application) to enter the participants’ responses using the participant’s unique study ID. The use of REDCap significantly reduces errors in data collection and the amount of missing data. REDCap will be accessed using university iPads, desktop computers or encrypted laptops dedicated to the study. The digitally recorded qualitative interviews will be labeled with each participant’s study ID and uploaded to the secure password protected SharePoint site. The transcriptionist will also have access to this site and will upload all transcripts to this site as well. The Research Coordinator and site PI’s will also have access to this site. There will be no sharing of individual data because of consent procedures.

### Data analysis

All data will be cleaned and checked for completeness and accuracy. Qualitative data from the interviews will be transcribed verbatim by an experienced transcriptionist.

#### Outcome analysis

Outcome analysis will include descriptive statistical analysis of data as well as analysis to understand the effect of MT4C-In Care on primary and secondary outcomes. To assess the impact of MT4C-In Care on the MCS, the primary outcome, we will use generalized estimating equations (GEE), and will run models using PROC GENMOD in SAS Version 9.3 [42]. The SF-12v2 MCS score is a continuous variable. Variables included in the GEE model will be group (intervention, control), time (baseline, 2 months, 4 months), and a group x time interaction term, which tests the hypothesis that MT4C-In Care promotes better mental health at 2 months and 4 months in the intervention as compared to the control group [43]. If a significant intervention effect is detected in this model, additional covariates (e.g., age, gender) will be added to adjust for statistically significant baseline differences observed in demographic characteristics between the intervention and control group. To model time, we will plot the mean MCS values in each group at each time period. Time will be included as either a continuous variable (if the plot suggests a linear trend) or a categorical variable (if the plot suggests a non-linear trend).

A key advantage of GEE, as compared to linear regression, is that no assumption is made that data are normally distributed. SAS allows the user to choose the most appropriate distribution and link function. In this study, we will select link function that is a best fit for the distribution of the participants’ MCS scores. If we observe a negative skew in MCS scores (as is the case in many populations), we will use a reflected transformation which can normalize the data [44], allowing for use of the identify link function which is used with a normal distribution.

While outcome analysis will follow the intent to treat principle, sensitivity analyses will also be completed to assess impact of the intervention upon outcomes with level of use as a covariate. The aim of the latter analysis is to explore whether amount of use of MT4C-In Care influences the impact of the intervention upon outcomes.

GEE assumes that data are missing completely at random [43]. This is difficult to verify [45], and data missingness more likely reflects a continuum from missing at random to missing not at random [46]. Despite this, experts recommend basing the primary analysis on the missing at random assumption [47]. We will use multiple imputation to handle missing data and will then analyze each dataset using GEE to determine the best method for handling missing data.

Analyses of secondary outcomes (hope, self-efficacy, grief, loneliness, and social support) will follow the same approach as for the primary outcome. An appropriate method for handling missing data for analysis of secondary outcomes will be selected based on observed missing patterns.

#### Process analysis

The purpose of the process analysis is to help explore and explain how participant demographic characteristics may influence study processes (e.g., recruitment), and how these study processes may have influenced the effectiveness of MT4C-In Care. Descriptive statistics will be used to calculate rates for recruitment, withdrawal and missing data. Descriptive statistics will summarize the self-reported frequency of use of difference sections of the MT4C-In Care, and will be used to calculate the proportion that perceived a change to knowledge and skills, mental health, and wellbeing.

#### Qualitative analysis

Sandelowski’s qualitative description method will guide the analysis of qualitative data [48], data collected from the sub-sample of participants during the 2-month qualitative interview to further evaluate MT4C-In Care and to inform the quantitative findings. NVivo 12 software will be used to manage and store the data. Each transcript will be read, looking for similarities, differences, and patterns in the data. These patterns will then be labelled as codes that will then grouped into themes. Trustworthiness of the data will be maintained by: 1) word for word transcriptions that will be checked by reviewing audio files, 2) ensuring that codes are data-driven and 3) audit trails will be kept to document analysis decisions through a coding journal. These steps will support transferability and confirmability. Qualitative data will be integrated with quantitative data at the results stage where similarities and differences will be explored. Differences between men and women, cultural majority and minority, older and younger carers, and carers of people with moderate and severe dementia will be explored after initial qualitative analysis has been completed.

### Dissemination

Each site has a stakeholder advisory committee comprised of community agencies, carers health system representatives and policy makers. A knowledge translation plan is being developed by each of these advisory committees to disseminate the findings in multiple formats such as carer stories, videos and summaries of the results. One page lay summaries of the results will also be available on community organization websites and a public end of grant knowledge translation event is being planned. Traditional dissemination will also occur through publications and presentations at conferences. An authorship guideline has been developed and agreed upon by the research team and advisory committee.

## Discussion

MT4C-In Care has been shown to be feasible and acceptable and holds promise in promoting mental well-being by supporting carers of a person living with dementia residing in LTC [24]. The study protocol outlined in this manuscript includes an assessment of the potential for MT4C-In Care to target social support and loneliness as well as hope, general self-efficacy and grief, which evidence supports are closely related to mental health.

Our design is a mixed methods pragmatic randomized controlled trial which also includes a mixed methods process evaluation. The findings from the process evaluation data will inform the outcome data findings as they will indicate to us if there are groups that seem to require further adaptation to optimize MT4C-In Care to their specific needs. The qualitative data will also inform the quantitative data, as it will help us to further evaluate MT4C-In Care by identifying the most important aspects and what potentially needs to be changed. Both the process data as well as the qualitative interview data will help explain statistically significant and non-significant findings related to effectiveness of MT4C-In Care in addressing the range of outcomes.

MT4C-In Care will be evaluated in four Canadian provinces. These different settings will add to our understanding of the effectiveness of MT4C-In Care as well as promote its ongoing use across Canada through the involvement of advisory committees. Once the study is completed, access will be open to the public for its use as one strategy to improve the mental health of carers of persons living with dementia residing in LTC. As well a francophone version will be developed and tested for feasibility.

Carers of persons living with dementia residing in LTC face many challenges such as working with staff and dealing with end of life issues [49]. They experience multiple concurrent transitions such as changing their roles and relationship with the person in LTC. This study is important as it will evaluate the effects of a web-based intervention to support carers to deal with their challenges and transitions.

## Supplementary information

**Additional file 1.** SPIRIT Checklist. Checklist for content in the protocol using the SPIRIT guidelines for reporting protocols.

**Additional file 2.** Consent forms: The two consent forms used in this study.

**Additional file 3.** My Tools 4 Care –In Care Checklist: Using this checklist participants will provide information on the use of My Tools 4 Care-In Care and evaluation of whether their knowledge was increased with use of the intervention.

**Additional file 4.** Qualitative Interview Guide: Interview guide with open ended questions to evaluated My Tools 4 Care- In Care.

## Data Availability

Not Applicable as this article reports the protocol for a study.

## Abbreviations

CONSORT: Consolidated Standards for Reporting Clinical Trials
GEE: General Estimating Equations
GSES: General Self Efficacy Scale
HHI: Herth Hope Index
MCS: Mental Component Summary
MT4C – In Care: My Tools 4 Care – In Care
MT4C: My Tools 4 Care
PCS: Physical Component Summary
SF-12v2: Short Form 12 version 2
