# Supplementary material for: Study protocol: pragmatic randomized control trial of my tools 4 care- in care (MT4C-in care) a web-based tool for family Carers of persons with dementia residing in long term care
Source: BMC Geriatr. 2020 Aug 10;20:285. doi: 10.1186/s12877-020-01690-w (PMC7418203; doi:10.1186/s12877-020-01690-w)
Supplement: Supplementary file 2 — Additional file 2. Consent forms: The two consent forms used in this study. [file 12877_2020_1690_MOESM2_ESM.docx]

**Letter of Information and Consent Form for Family Caregiver Participants (intervention)**

**Study Title:** Supporting Family Caregivers of Persons Living with Dementia

**Principal Investigators:** Dr. Wendy Duggleby

Faculty of Nursing, University of Alberta

Edmonton, Alberta

780-492-8660

[wendy.duggleby@ualberta.ca](mailto:livingwithhope@nurs.ualberta.ca)

Dr. Hannah O’Rourke

Faculty of Nursing, University of Alberta

Edmonton, Alberta

780-492-7961

hannah.orourke@ualberta.ca

**Research Sponsor**: Public Health Agency of Canada

**Background**:

You are being asked to take part in this study because you are a caregiver for a family member or friend who has dementia and is living in a long term care facility. This Information and Consent Form is part of the process of informed consent. It explains this research study and what will happen to you if you choose to be in this study. If you would like to know more about anything you read here, or have any questions at any time regarding this research study, please be sure to ask the researchers or research assistant. Read this form carefully to make sure you understand all the information provided. You will get a copy of this form to keep. You do not have to take part in this study if you don’t want to. Your family member’s care does not depend on whether or not you take part.

**Purpose of the Study:**

The purpose of this study is to help us evaluate a new way of supporting family caregivers. We want to know how effective the approach is, and how it may influence factors such as your hope, quality of life, loss and grief, loneliness, social support and ability to deal with adverse situations. The overall goal of this research is to help caregivers like you.

**Procedure:**

Approximately 280 people like you will take part in this study (100 in Alberta, 100 in Ontario, 40 in Saskatchewan and 40 in Manitoba). If you agree to take part in this study, you will be asked to talk with a researcher by telephone for about 30 minutes to complete the surveys at three time points (first survey, 2 months, and 4 months). The researcher will ask questions about you such as your age and that of your family member/friend. You will also be asked questions about your hope, quality of life, loss and grief, loneliness, social support and ability to deal with difficult situations. All interviews will be audio-taped.

If you take part in the study, you will be randomly assigned to one of two different ways of supporting family caregivers. Both ways will involve receiving a copy of an educational booklet from the Alzheimer Society.

Note that there is an equal chance of being assigned to either group. You will not know to which group you have been assigned. Knowing more about the two groups may affect the results of the study. At the end of the study, you will be given the opportunity to receive the approach that the other group had, and to ask any questions.

You will be given instructions on how to access an online toolkit (My Tools 4 Care – In Care) for you to use as you wish for 2 months. The information that you add to this online toolkit will be confidential. After two months you will be asked questions from a checklist on your use of the toolkit through the completion of a survey administered over the phone. You may also be asked to participate in a short interview to tell us more about your experiences with using the toolkit.

In recognition of your time for this study, you will be given a $20.00 coffee gift card. Even though you are receiving this gift, you do not need to answer any questions you don’t want to.

There is no cost to you associated with taking part in the study other than your time which will be at the most 2 hours over a 4 month time period, plus time spent on the online toolkit.

Below is a table of the timing of the surveys that you will be asked to complete:

|  | Initial | Two months | Four months |
| --- | --- | --- | --- |
| Demographic (5min) | x |  |  |
| Hope (5min) | x | x | x |
| Quality of Life (5 min) | x | x | x |
| Self-Efficacy (5 min) | x | x | x |
| Loss and Grief (5 min) | x | x | x |
| Loneliness (1 min) | x | X | x |
| Social Support (5 min) | x | x | x |
| Toolkit | x |  |  |
| Interview (15-30 minutes) |  | x |  |
| Toolkit Checklist (10 min) |  | x |  |

**Potential Risks:**

There are minimal risks to taking part in this study. It is possible you may feel distressed or upset during the interview because talking about your experiences may make you feel sad. You are free to stop an interview or stop taking part in the study at any time, just notify the research assistant. If you want, the research assistant may offer to refer you to someone who is not directly involved with the study, for you to talk to.

**Potential Benefits:**

The research may benefit you directly, as using the online toolkit may increase your hope. You may also find it helpful to talk about your experience. The results of the research will help us find out the best way to help caregivers like you.

**Confidentiality:**

Every effort will be made to keep confidential any information that is obtained during this research study. The information collected will not have your name on it. All reporting will be done in a group format, so you will not be able to be identified. Anything that we find out about you that could identify you will not be published or told to anyone. Your identity will remain protected in any publications or presentations of the study results.

What you tell us will be stored in a locked filing cabinet and in a secure place on a computer. Only the research team will see the data and that will not have your name on it.

The data will be kept in a locked cabinet of one of the Principal Investigators (Wendy Duggleby and Hannah O’Rourke) for a period of five years following the completion of the study. The findings will be published in scholarly journals as well as presented at various conferences related to caregiving.

**Participation and Withdrawal:**

Your participation in this study is voluntary. If you decide to take part in the study, you are free to stop at any time, even after signing the consent form or part-way through the study. If you decide to stop taking part in the study, there will be no penalty to yourself. Just let the researcher know. If you do not want to answer some of the questions you do not have to but you can still be in the study. If you would like your data to be destroyed, just let us know. The study data may be used again in a future study and if so, ethics approval will be obtained.

**Study Debriefing:**

You may obtain information about the results of the study by indicating so on the consent form. The research findings will be sent via e-mail or by mail to the address you provide.

**Rights of Research Participants:**

You may withdraw your consent at any time and discontinue participation without penalty. You are not waiving any legal claims, rights or remedies because of your participation in this research study. This study has been reviewed and received ethics clearance through the University of Alberta Health Research Ethics Board. If you have questions regarding your rights as a research participant, contact:

Health Research Ethics Board Administration Office
308 Campus Tower, 8625-112 Street
Edmonton, AB T6G 1K8
Phone: 780-492-2615

**CONTACT NAMES AND TELEPHONE NUMBERS:**

Please contact the individuals identified below, at the time of consent and at any time during the study, if you have any questions or concerns about the research project and procedures:

**Principal Investigators:** Wendy Duggleby, PhD, RN, University of Alberta ([wendy.duggleby@ualberta.ca](mailto:wendy.duggleby@ualberta.ca); 780-492-8660) or Hannah O’Rourke, PhD, RN, University of Alberta (hannah.orourke@ualberta.ca; 780-492-7961)

# CONSENT FORM

**Study Title:** Supporting Family Caregivers of Persons Living with Dementia

**Principal Investigators** Dr. Wendy Duggleby

Faculty of Nursing, University of Alberta

Edmonton, Alberta, Canada

780-492-8660

[wendy.duggleby@ualberta.ca](mailto:livingwithhope@nurs.ualberta.ca)

Dr. Hannah O’Rourke

Faculty of Nursing, University of Alberta
Edmonton, Alberta
780-492-7961

hannah.orourke@ualberta.ca

|  |  | **Yes** | **No** |
| --- | --- | --- | --- |
|  | Do you understand that you have been asked to be in a research study? | ❑ | ❑ |
|  | Have you read and received a copy of the attached Information Sheet? | ❑ | ❑ |
|  | Do you understand the benefits and risks involved in taking part in this research study? | ❑ | ❑ |
|  | Have you had an opportunity to ask questions and discuss this study? | ❑ | ❑ |
|  | Do you understand that you are free to leave the study at any time, without  having to give a reason and without affecting your loved one’s medical care? | ❑ | ❑ |
|  | Has the issue of confidentiality been explained to you? | ❑ | ❑ |
|  | Do you understand who will have access to your data, including personally identifiable information? | ❑ | ❑ |
| Do you know your speech will be audio-recorded and will be used for research purposes? | | ❑ | ❑ |
|  | Who explained this study to you? ________________________________________________ |  |  |
|  | I agree to take part in this study. | ❑ | ❑ |

***Oral consent of research participant:***

_______ ______________________________

Name of Participant Date

***Signature of Person Obtaining Consent:*** I believe that the participant understands what is involved in the study and voluntarily agrees to take part in.

________ ____________________

Name of Person Obtaining Consent (please print) Signature of Person Obtaining Consent Date

**Letter of Information and Consent Form for Family Caregiver Participants (Control)**

**Study Title:** Supporting Family Caregivers of Persons Living with Dementia

**Principal Investigators:** Dr. Wendy Duggleby

Faculty of Nursing, University of Alberta

Edmonton, Alberta

780-492-8660

[wendy.duggleby@ualberta.ca](mailto:livingwithhope@nurs.ualberta.ca)

Dr. Hannah O’Rourke

Faculty of Nursing, University of Alberta
Edmonton, Alberta

780-492-7961

hannah.orourke@ualberta.ca

**Research Sponsor**: Public Health Agency of Canada

**Background**:

You are being asked to take part in this study because you are a caregiver for a family member or friend who has dementia and is living in a long term care facility. This Information and Consent Form is part of the process of informed consent. It explains this research study and what will happen to you if you choose to be in this study. If you would like to know more about anything you read here, or have any questions at any time regarding this research study, please be sure to ask the researchers or research assistant. Read this form carefully to make sure you understand all the information provided. You will get a copy of this form to keep. You do not have to take part in this study if you don’t want to. Your family member’s care does not depend on whether or not you take part.

**Purpose of the Study:**

The purpose of this study is to help us evaluate a new way of supporting family caregivers. We want to know how effective the approach is, and how it may influence factors such as your hope, quality of life, loss and grief, loneliness, social support and ability to deal with adverse situations. The overall goal of this research is to help caregivers like you.

**Procedure:**

Approximately 280 people like you will take part in this study (100 in Alberta, 100 in Ontario, 40 in Saskatchewan and 40 in Manitoba). If you agree to take part in this study, you will be asked to talk with a researcher by telephone for about 30 minutes to complete the surveys at three time points (first survey, 2 months, and 4 months). The researcher will ask questions about you such as your age and that of your family member/friend. You will also be asked questions about your hope, quality of life, loss and grief, loneliness, social support and ability to deal with difficult situations. All interviews will be audio-taped.

If you take part in the study, you will be randomly assigned to one of two different ways of supporting family caregivers. Both ways will involve receiving a copy of an educational booklet from the Alzheimer Society.

Note that there is an equal chance of being assigned to either group. You will not know to which group you have been assigned. Knowing more about the two groups may affect the results of the study. At the end of the study, you will be given the opportunity to receive the approach that the other group had, and to ask any questions.

In recognition of your time for this study, you will be given a $20.00 coffee gift card. Even though you are receiving this gift, you do not need to answer any questions you don’t want to.

There is no cost to you associated with taking part in the study other than your time which will be at the most 1.5 hours over a 4 month time period.

Below is a table of the timing of the surveys that you will be asked to complete:

|  | Initial | Two months | Four months |
| --- | --- | --- | --- |
| Demographic (5min) | x |  |  |
| Hope (5min) | x | x | x |
| Quality of Life (5 min) | x | x | x |
| Self-Efficacy (5 min) | x | x | x |
| Loss and Grief (5 min) | x | x | x |
| Loneliness (1 min) | x | X | x |
| Social Support (5 min) | x | x | x |

**Potential Risks:**

There are minimal risks to taking part in this study. It is possible you may feel distressed or upset during the interview because talking about your experiences may make you feel sad. You are free to stop an interview or stop taking part in the study at any time, just notify the research assistant. If you want, the research assistant may offer to refer you to someone who is not directly involved with the study, for you to talk to.

**Potential Benefits:**

The research may benefit you directly, as you may find it helpful to talk about your experience. The results of the research will also help us find out the best way to help caregivers like you.

**Confidentiality:**

Every effort will be made to keep confidential any information that is obtained during this research study. The information collected will not have your name on it. All reporting will be done in a group format, so you will not be able to be identified. Anything that we find out about you that could identify you will not be published or told to anyone. Your identity will remain protected in any publications or presentations of the study results.

What you tell us will be stored in a locked filing cabinet and in a secure place on a computer. Only the research team will see the data and that will not have your name on it.

The data will be kept in a locked cabinet of one of the Principal Investigators (Wendy Duggleby and Hannah O’Rourke) for a period of five years following the completion of the study. The findings will be published in scholarly journals as well as presented at various conferences related to caregiving.

**Participation and Withdrawal:**

Your participation in this study is voluntary. If you decide to take part in the study, you are free to stop at any time, even after signing the consent form or part-way through the study. If you decide to stop taking part in the study, there will be no penalty to yourself. Just let the researcher know. If you do not want to answer some of the questions you do not have to but you can still be in the study. If you would like your data to be destroyed, just let us know. The study data may be used again in a future study and if so, ethics approval will be obtained.

**Study Debriefing:**

You may obtain information about the results of the study by indicating so on the consent form. The research findings will be sent via e-mail or by mail to the address you provide.

**Rights of Research Participants:**

You may withdraw your consent at any time and discontinue participation without penalty. You are not waiving any legal claims, rights or remedies because of your participation in this research study. This study has been reviewed and received ethics clearance through the University of Alberta Health Research Ethics Board. If you have questions regarding your rights as a research participant, contact:

Health Research Ethics Board Administration Office
308 Campus Tower, 8625-112 Street
Edmonton, AB T6G 1K8
Phone: 780-492-2615

**CONTACT NAMES AND TELEPHONE NUMBERS:**

Please contact the individuals identified below, at the time of consent and at any time during the study, if you have any questions or concerns about the research project and procedures:

**Principal Investigators:** Wendy Duggleby, PhD, RN, University of Alberta ([wendy.duggleby@ualberta.ca](mailto:wendy.duggleby@ualberta.ca); 780-492-8660) or Hannah O’Rourke, PhD, RN, University of Alberta (hannah.orourke@ualberta.ca; 780-492-7961)

# CONSENT FORM

**Study Title:** Supporting Family Caregivers of Persons Living with Dementia

**Principal Investigators:** Dr. Wendy Duggleby

Faculty of Nursing, University of Alberta

Edmonton, Alberta, Canada

780-492-8660

[wendy.duggleby@ualberta.ca](mailto:livingwithhope@nurs.ualberta.ca)

Dr. Hannah O’Rourke

Faculty of Nursing, University of Alberta

Edmonton, Alberta

780-492-7961

hannah.orourke@ualberta.ca

|  |  | **Yes** | **No** |
| --- | --- | --- | --- |
|  | Do you understand that you have been asked to be in a research study? | ❑ | ❑ |
|  | Have you read and received a copy of the attached Information Sheet? | ❑ | ❑ |
|  | Do you understand the benefits and risks involved in taking part in this research study? | ❑ | ❑ |
|  | Have you had an opportunity to ask questions and discuss this study? | ❑ | ❑ |
|  | Do you understand that you are free to leave the study at any time, without  having to give a reason and without affecting your loved one’s medical care? | ❑ | ❑ |
|  | Has the issue of confidentiality been explained to you? | ❑ | ❑ |
|  | Do you understand who will have access to your data, including personally identifiable information? | ❑ | ❑ |
| Do you know your speech will be audio-recorded and will be used for research purposes? | | ❑ | ❑ |
|  | Who explained this study to you? ________________________________________________ |  |  |
|  | I agree to take part in this study. | ❑ | ❑ |

***Oral consent of research participant:***

_______ ______________________________

Name of Participant Date

***Signature of Person Obtaining Consent:*** I believe that the participant understands what is involved in the study and voluntarily agrees to take part in.

________ ____________________

Name of Person Obtaining Con
