# Supplementary material for: Study protocol: pragmatic randomized control trial of my tools 4 care- in care (MT4C-in care) a web-based tool for family Carers of persons with dementia residing in long term care
Source: BMC Geriatr. 2020 Aug 10;20:285. doi: 10.1186/s12877-020-01690-w (PMC7418203; doi:10.1186/s12877-020-01690-w)
Supplement: Supplementary file 3 — Additional file 3. My Tools 4 Care –In Care Checklist: Using this checklist participants will provide information on the use of My Tools 4 Care-In Care and evaluation of whether their knowledge was increased with use of the intervention. [file 12877_2020_1690_MOESM3_ESM.docx]

**MT4C-InCare Checklist**

1. How often did you use My Tools 4 Care – In Care over the last 2 months?

| *Never* | *Daily* | *2-3 times/week* | *4-6 times/week* | *Weekly* | *Monthly* | *Other* |
| --- | --- | --- | --- | --- | --- | --- |
| ❑ | ❑ | ❑ | ❑ | ❑ | ❑ | _______________ |

1. What was the total time you spent on MT4C-InCare over the past 2 months?
2. What parts of MT4C-In Care did you use and which part did you like the best?

| *Section* | *I used this  section:* | | *My favorite Section (check one or more)* |
| --- | --- | --- | --- |
|  | Yes | No |  |
| Section 1 About Me: Where I am | ❑ | ❑ |  |
| Section 1 About Me: What helps me? | ❑ | ❑ |  |
| Section 1 About Me: My goals as a care partner | ❑ | ❑ |  |
| Section 1 About Me: Everyday Hope | ❑ | ❑ |  |
| Section 1 About Me: What am I doing for myself today? | ❑ | ❑ |  |
| Section 1 About Me: How can I manage the guilt that I feel? | ❑ | ❑ |  |
| Section 1 About Me: Working together | ❑ | ❑ |  |
| Section 1 About Me: Advocating for care | ❑ | ❑ |  |
| Section 1 About Me: Tips for Visits | ❑ | ❑ |  |
| Section 1 About Me: Goals for care at the end of life | ❑ | ❑ |  |
| Section 1 About Me: Building your network | ❑ | ❑ |  |
| Section 2: Common changes to expect | ❑ | ❑ |  |
| Section 3: Frequently Asked Questions | ❑ | ❑ |  |
| Section 4: Resources | ❑ | ❑ |  |

1. Please tell us whether you agree or disagree with the following statements:

|  |  | *Strongly Disagree* | *Disagree* | *Neutral* | *Agree* | *Strongly Agree* |
| --- | --- | --- | --- | --- | --- | --- |
|  | The directions were clear for each activity I wanted to do. | ❑ | ❑ | ❑ | ❑ | ❑ |
|  | I was sure about what to do with each activity I wanted to do. | ❑ | ❑ | ❑ | ❑ | ❑ |
|  | I had enough energy to complete each activity I wanted to do. | ❑ | ❑ | ❑ | ❑ | ❑ |
|  | I had enough time to complete each activity I wanted to do. | ❑ | ❑ | ❑ | ❑ | ❑ |
|  | I was able to complete all the activities I wanted to do. | ❑ | ❑ | ❑ | ❑ | ❑ |
|  | MT4C-In Care increased my ability to deal with significant changes. | ❑ | ❑ | ❑ | ❑ | ❑ |
|  | I would recommend MT4C-InCare to someone else. | ❑ | ❑ | ❑ | ❑ | ❑ |
|  | The online format of MT4C-In Care is easy to use. | ❑ | ❑ | ❑ | ❑ | ❑ |
|  | The online format of MT4C–In Care is convenient for me. | ❑ | ❑ | ❑ | ❑ | ❑ |

1. Please tell us how satisfied you were with the toolkit by circling the number below that best represents your satisfaction:

1 (dissatisfied) 2(satisfied) 3 (greatly satisfied)

1. Would you say that:
   1. Your knowledge and skills have improved as a result of MT4C-In Care ❑ Yes ❑ No
   2. Your mental health has improved as a result of MT4C-In Care ❑ Yes ❑ No
   3. Your wellbeing has improved as a result of MT4C-In Care ❑ Yes ❑ No
2. Any other comments?
