# Supplementary material for: Study protocol: pragmatic randomized control trial of my tools 4 care- in care (MT4C-in care) a web-based tool for family Carers of persons with dementia residing in long term care
Source: BMC Geriatr. 2020 Aug 10;20:285. doi: 10.1186/s12877-020-01690-w (PMC7418203; doi:10.1186/s12877-020-01690-w)
Supplement: Supplementary file 4 — Additional file 4. Qualitative Interview Guide: Interview guide with open ended questions to evaluated My Tools 4 Care- In Care. [file 12877_2020_1690_MOESM4_ESM.docx]

**Evaluation of MT4C-InCare Interview Guide for Caregivers**

**Code no: ______________ Date: ______________________ Visit: ___________________**

1. What were you thinking about when you worked on MT4C-InCare?
2. Did it help you deal with your significant changes? Why or Why not?
3. Did anything influence your ability to work on MT4C-InCare?
4. Who do you think would benefit most from MT4C-InCare?
5. What did you like best?
6. What did you like least?
7. Anything else you would like to add?
